# The Senescence Markers p16INK4A, p14ARF/p19ARF, and p21 in Organ Development and Homeostasis

**DOI:** 10.3390/cells11121966

**Published:** 2022-06-19

**Authors:** Kay-Dietrich Wagner, Nicole Wagner

**Affiliations:** CNRS, INSERM, iBV, Université Côte d’Azur, 06107 Nice, France

**Keywords:** development, aging, endothelial cells, senescence, SASP, metabolic function, stem cells

## Abstract

It is widely accepted that senescent cells accumulate with aging. They are characterized by replicative arrest and the release of a myriad of factors commonly called the senescence-associated secretory phenotype. Despite the replicative cell cycle arrest, these cells are metabolically active and functional. The release of SASP factors is mostly thought to cause tissue dysfunction and to induce senescence in surrounding cells. As major markers for aging and senescence, p16INK4, p14ARF/p19ARF, and p21 are established. Importantly, senescence is also implicated in development, cancer, and tissue homeostasis. While many markers of senescence have been identified, none are able to unambiguously identify all senescent cells. However, increased levels of the cyclin-dependent kinase inhibitors p16INK4A and p21 are often used to identify cells with senescence-associated phenotypes. We review here the knowledge of senescence, p16INK4A, p14ARF/p19ARF, and p21 in embryonic and postnatal development and potential functions in pathophysiology and homeostasis. The establishment of senolytic therapies with the ultimate goal to improve healthy aging requires care and detailed knowledge about the involvement of senescence and senescence-associated proteins in developmental processes and homeostatic mechanism. The review contributes to these topics, summarizes open questions, and provides some directions for future research.

## 1. Introduction

Senescence was first described by Hayflick in isolated fibroblasts in culture [1,2]. In response to repeated replication, DNA damage, metabolic alterations, reactive oxygen species or cytotoxic drugs, cells enter permanent cell cycle arrest, change their morphology to more flat and large cells, express and secrete cytokines, chemokines, growth factors, bioactive lipids, and pro-apoptotic factors—the so-called senescence-associated secretory phenotype (SASP) and become positive for senescence-associated beta-galactosidase (SAβG) [3,4,5,6,7,8,9,10,11]. Although the morphological features are easy to follow in cultured cells, the identification in vivo or on histological sections is more problematic. SAβG staining is also not uniform in all old cells or in response to typical inducers of senescence, e.g., doxorubicin [12]. During embryonic development, even some co-localization of SAβG staining with proliferation markers was detectable [13]. Thus, recently the use of combinations of different markers and expression of SASP factors was suggested from the International Cell Senescence Association to correctly identify senescent cells [5]. In addition, the expression of SASP factors varies depending on different cell types [14]. Whether different cell types are to the same extent susceptible to age-related senescence is equally unclear. The conventional view in agreement with the Hayflick experiments would suggest that replicative cells are prone to senescence with increasing age. Nevertheless, senescence-like features were also observed in terminally differentiated non-cycling cells [15,16,17,18] and in macrophages and T-cells [19,20,21]. As typical markers for aging and senescence p16INK4A, p14ARF/p19ARFArf, and p21 are accepted [3,4,5,6,7,10,11,22,23,24,25,26,27,28]. These proteins were originally identified as cell cycle inhibitors (for details see below). Thus, senescence could also be viewed as an extreme case of cell cycle inhibition except for the case of postmitotic cells. p16INK4A is one of the most attractive and intensively investigated marker of aging and senescence as expression has been initially reported to be absent during embryonic development [29,30] and it is highly expressed in advanced age and senescence [24,25,26,27,28,31,32,33,34,35,36,37]. We and others provided recent evidence that p16INK4A is expressed during development in several organs [38]. The elimination of p16INK4A-expressing cells in aged animals did not only have the expected positive effects, but also negatively impacted the health span, caused liver fibrosis [39] and interfered with normal wound healing [40,41]. Thus, it seems timely to review knowledge of senescence, p16INK4A, p14ARF/19ASRF, and p21 in embryonic and postnatal development, in disease and homeostasis.

## 2. p16INK4A, p14ARF/p19ARF, and p21—Basic Molecular Mechanisms

*p16INK4A* was originally identified as a tumor suppressor gene [42,43]. Initially, different names, i.e., multiple tumor suppressor-1 (MTS-1), inhibitor of cyclin dependent kinase 4a (INK4a), cyclin dependent kinase inhibitor 2a (CDKN2A), have been used, *CDKN2A* now being the official gene symbol. The human *p16INK4A* gene is located on the short arm of chromosome 9 (9p21.3) while the mouse gene is located on chromosome 4. The use of different open reading frames on the locus generates in both species’ alternative proteins (p14p14ARF in humans and p19ARF in mice). In comparison to p16INK4A, they differ in the first exon while they share the second exon, resulting in the translation of different reading frames [44,45] (reviewed in [46]). The *p21* gene (*CDKN1A*) is completely independent and localized on chromosomes 6 and 17 in humans and mice, respectively. p16INK4A acts as a specific inhibitor of the cyclin-dependent kinases CDK4 and CDK6 that is mainly active in the G1 phase of the cell cycle to prevent the cell transition from the G1 to S phase and subsequent proliferation arrest by rendering retinoblastoma protein (pRB) in a hypo-phosphorylated state. CDK 4/6 bind cyclin D to form a complex that phosphorylates retinoblastoma protein. When phosphorylated, pRB dissociates from E2F transcription factors which translocate to the nucleus and activate transcription of S phase genes which results in a cellular proliferation [47,48,49]. p16INK4A expression is tightly regulated via a negative feedback loop with pRB. pRB phosphorylation promotes E2F translocation and induces p16INK4A expression, which in turn inhibits CDK 4/6 and increases hypo-phosphorylated pRB, leading to the downregulation of p16INK4A [50]. Alternatively, elevated p16INK4A transcription in pRB negative cells has also been reported, indicating alternative mechanisms for p16INK4A upregulation [25]. Furthermore, differences in p16INK4A RNA expression did not correlate well with the pRB status of the cells [25]. p16INK4A and p19p14ARF/p19ARF are suppressed by promoter hypermethylation via PRC1 and PRC2 complexes involving BMI-1, EZH2, ZFP 277, Mel18, CXB7, and CXB8 proteins [51,52,53,54,55,56,57,58,59,60,61]. Interestingly, pRB seems to also be involved in this regulatory loop as a lack of pRB results in loss of histone H3K27 trimethylation and less recruitment of BMI-1 and repression of the p16INK4A locus [62]. Activators of the p16INK4A locus include AP-1 [63], JDP-2 [64,65,66], CTCF [67], Tcf-1 [68], p300 with Sp-1 [69], Meis1 [70], and PPAR gamma [71]. These in vitro molecular studies should be interpreted with care. For example, multiple beneficial effects were attributed to removal of p16INK4A-expressing senescent cells in mice [17,72,73,74,75,76,77,78,79,80,81,82]. PPAR gamma stimulation induces p16INK4A-expression and might result in senescent cell-based multi-organ failure. However, glitazones (PPAR gamma activators, e.g., rosiglitazone) have been in clinical use as antidiabetic drugs for more than 20 years [83].

Combined in vivo and in vitro studies using knockout mouse models, chromatin immunoprecipitation (CHIP), and RNA sequencing showed that non-cleaved general transcription factor TFIIA acts as a repressor of the p16INK4A, p14ARF/p19ARF, and p21 loci. Taspase1-mediated (TASP1-mediated) cleavage of TFIIA ensures rapid cell proliferation and morphogenesis by reducing transcription of p16INK4A and p14ARF/p19ARF. Consequently, Tasp1 knockout mice showed variable degrees of micro-ophthalmia, anophthalmia, agnathia, general growth retardation, and defects of development of forebrain neurons, which were partially rescued by combined knockout of p16INK4A and p14ARF/p19ARF [84].

Elegant in vivo studies showed that a common variant of a 58 kb non-coding sequence in humans (70 kb in mice) flanking the p16INK4A/p14ARF/p19ARF locus is associated with an increased risk of coronary artery disease [85,86,87]. The removal of this sequence resulted in a low expression of p16INK4A/p14ARF/p19ARF and excessive proliferation of aortic smooth muscle cells indicating that this region has a pivotal role in the regulation of p16INK4A/p14ARF/p19ARF expression and control of cell proliferation [88].

Coordinated suppression/activation of the p16INK4A/p14ARF/p19ARF locus would further implicate that p16INK4A and p14ARF/p19ARF expression patterns are related. Our recent study on several organs during development and aging showed that this is not the case [38]. Moreover, p14ARF/p19ARF shows different downstream signaling from p16INK4A. p14ARF/p19ARF acts as a cell cycle inhibitor by interfering with the activation of the P53 pathway, through binding to MDM2 and blocking MDM2-mediated P53 degradation [89], although p14ARF/p19ARF might also have some P53-independent actions [90]. p14ARF/p19ARF might induce apoptosis via Bax in a P53 independent manner [91]. p14ARF/p19ARF is activated by Myc [92] and in a feedback mechanism seems to physically interact with Myc protein and prevent its function as a transcriptional activator. In addition, this action is independent of P53 [93,94]. p21 is another cyclin-dependent kinase inhibitor and has been shown to fulfill anti-proliferative functions in a mechanism that is P53-dependent. p16INK4A might activate p21, which acts by inhibiting CDK2-cyclin E active complex formation, such as p16INK4A inhibition of CDK4/6 cyclin D. The consequence is also hypo-phosphorylation of pRB and cell cycle arrest [95]. Interesting, low p21 levels promote CDK-cyclin complex formation, while higher expression of p21 inhibits the activity of the complex [96]. This might explain to some extent the diverse effects of altering the levels in vivo described below.

pRB interacts through various cellular proteins, among which E2F transcription factors are the best characterized [97,98,99]. While transient E2F overexpression promotes cell cycle progression and hyperplasia, sustained E2F3 overexpression promotes a senescence-like phenotype in a p16INK4A-pRB-p14ARF/p19ARF pathway-dependent manner [100] points again to the different outcomes dependent on timing and cellular model. E2F3 in addition occupies the p14ARF/p19ARF promoter in mouse embryonic fibroblasts and E2f3 loss is sufficient to derepress p14ARF/p19ARF, which in turn triggers activation of p53 and expression of p21 [101]. The combined loss of all E2F transcription factors also results in overexpression of p21, leading to a decrease in cyclin-dependent kinase activity and Rb phosphorylation [98,99]. p21 is furthermore transcriptionally inhibited by a Myc-Miz complex [102,103] and activated by Smad/FoxO complexes in response to TGF beta stimulation [104]. The regulation of p16INK4A, p14/p14ARF/p19ARFArf, and p21 are reviewed in detail elsewhere [105,106,107,108,109,110].

## 3. p16INK4A, p14ARF/p19ARF, and p21 in Organ Development

Earlier studies were not able to detect p16INK4A expression during mouse embryonic development [29,30]. However, the authors did not exclude the possibility that p16INK4A might be expressed in different developing organs and time points, but the lack of p16INK4A detection might rather represent technical limits [29]. We used recently sensitive quantitative RT-PCR and immunohistochemistry techniques [111,112,113,114,115] to re-evaluate p16INK4A expression during mouse embryonic development, in the adult, and in old mice [38]. We determined p16INK4A expression between embryonic day (E10) and birth, at postnatal day seven (P7), postnatal day 21, which corresponds to weaning, in adults, and 16–18-month-old mice. We focused on the heart, brain, liver, and kidney as these organs or progenitors are already present at the first time point chosen [116,117,118,119]. p16INK4A, p14ARF/p19ARF, and p21 were detectable at all investigated embryonic and postnatal time points. Compared to p14ARF/p19ARF and p21, p16INK4A expression continued to increase during development, remained then stable in adulthood and became dramatically upregulated in the organs of old animals. This high rise of p16INK4A expression with old age is in principle in agreement with the literature defining p16INK4A as a marker of aging and senescence [5,72,120,121,122,123,124]. In agreement with this, we also detected a significant increase in SASP markers in all investigated organs of old animals. Interestingly, in the organs of old mice, we observed the highest p16INK4A expression in vascular structures, especially in the liver and the heart. During embryonic development, high p16INK4A expression did not correspond with increased SASP expression and was observed in the respective parenchymal cells and not in vessels, which coincided with the corresponding time points of differentiation in the organs investigated [38], suggesting that in this instance, p16INK4A might act in a classical way as cell cycle inhibitor as pre-requisite for differentiation. Although we did not yet identify potential molecular regulators of p16INK4A expression during embryonic development, it is interesting to note that p16INK4A and p14ARF/p19ARF displayed a differential developmental expression pattern indicating that not the genomic locus, but more specific regulatory elements of p16INK4A might be activated.

In contrast to the early reports of absent p16INK4A expression during mouse development [29,30], expression during rat brain development was described only slightly afterward. In agreement with our results, p16INK4A expression colocalized with p53 in the ventricular and subventricular zones at embryonic and early postnatal stages and p53 was mainly found in postmitotic cells of the cerebral cortex and hippocampus [125]. In the olfactory epithelium, p16INK4A and p21 were detectable around birth, with p16INK4A marking differentiating and p21 mature neurons [126]. p16INK4A expression was also detected in bone marrow derived hematopoietic progenitor cells of adults [127] and in epiphyseal growth plate chondrocytes and bone lining osteoblasts in growing mice [128]. In these cases, higher p16INK4A expression was associated with reduced cell proliferation, but senescence of these cells had not been reported. Increasing p16INK4A and p21 expression has been also observed in male germ cells coinciding with the timing of mitotic arrest, but not with senescence [129]. These male germ cells enter meiosis during post-natal life [130]. Increased p16INK4A expression was noted already in the endometrium between days 2 to 5 of pregnancy in mice. Injection of a p16INK4A antibody decreased the number of implanted blastocysts compared with that of a saline-injected group suggesting a role of p16INK4A in blastocyst implantation [131]. This observation seems to be in contrast to normal Mendelian frequencies of birth in p16INK4A knockout mice [132], but slight deviations from Mendelian inheritance might become obvious only when analyzing large numbers of pups [115] and implantation defects would be only detectable if the female mice in mating are p16INK4A knockout instead of heterozygotes. p16INK4A expression was also described in human endometrium during pregnancy [133].

During mouse embryonic development, p16INK4A was further detected in the gut in intestinal stem cells and progenitor compartments. Loss of Bmi1 resulted in accumulation of p16INK4A and p14ARF/p19ARF and reduced intestinal stem cell proliferation, which was accompanied by increased differentiation to the post-mitotic goblet cell lineage. Bmi1 expression in intestinal stem cells was co-regulated by Notch and beta-catenin [134]. Bmi1 plays also important roles for maintenance of neural stem cell self-renewal [135,136,137,138,139], for mesenchymal stem cell renewal and bone formation [140], for immature retinal progenitor/stem cells and retinal development [141], and for hepatic stem cell expansion [142] via negative regulation of p16INK4A, p14ARF/p19ARF, and p21.

Already in three-month-old mice, a significant number of p16INK4A-expressing cardiomyocytes, mostly bi- and multinucleated cells, had been described [143]. We investigated expression much earlier during embryonic development and found increased expression coinciding with cardiomyocyte differentiation [38]. As isolated cardiomyocytes were immunostained in the previously mentioned report, potential expression in endothelial cells at this time point was not detected. The authors considered p16INK4A expression as a marker of senescence without further approaches to identify the cells as senescent [143]. Another study investigated the proliferation of cardiomyocytes by PCNA staining ex vivo in p16INK4A/p14ARF/p19ARF knockout mice. Surprisingly, the authors report 70% of proliferating cardiomyocytes from 8 weeks old mice [144], which is in obvious contrast to all data in the literature.

Specific p16INK4A knockout mice which retained p14ARF/p19ARF function displayed an increased incidence of spontaneous and carcinogen-induced cancers [132] and melanomas [145] and thymus hyperplasia [132]. Thymus hyperplasia was associated with increased numbers of CD4 and CD8 lymphocytes, which was surprisingly not due to increased proliferation, but to reduced apoptosis of lymphocytes [146]. Mice lacking p16INK4A and p14ARF/p19ARF on an FVBN genetic background develop cataracts and micro-ophthalmia. They showed beginning from E15.5 defects in the developmental regression of the hyaloid vascular system, retinal dysplasia, abnormal differentiation of the lens, and cataracts [147]. Interestingly, the micro-ophthalmia phenotype in Task1 knockout mice was partially rescued by the lack of p16INK4A and p14ARF/p19ARF suggesting a fine-tuning of neuronal and eye development by the two proteins [84].

In addition, p14ARF/p19ARF knockout mice are prone to spontaneous and carcinogen-induced cancers [148]. p14ARF/p19ARF is involved in perivascular cell accumulation postnatally in the mouse eye before eye development is completed [147,149,150,151]. p14ARF/p19ARF decreased Pdgfr beta expression and blocked Pdgf-B-driven proliferation independently of Mdm2 and p53, which prevents the accumulation of perivascular cells and allows regression of the hyaloid vascular system of the developing eye [152,153]. Tgfbeta2 is required for p14ARF/p19ARF transcription in the hyaloid vascular system as well as in the cornea and the umbilical arteries [154,155].

p14ARF/p19ARF is detectable in developing hepatoblasts [156], which agrees with our recent report. Lack of the Tbx3 member of the T-box family of transcription factors results in upregulation of p14ARF/p19ARF and p21 in the developing liver, which is associated with severe defects in proliferation and in hepatobiliary lineage segregation, including the promotion of cholangiocyte differentiation and abnormal liver development [156]. Whether Tbx3 might directly regulate p14ARF/p19ARF and p21 expression was not determined in this study.

p21 knockout mice were reported initially to develop normally despite defective G1 checkpoint control in isolated knockout embryonic fibroblasts [157]. Interestingly, p21 expression was detected by Western Blot in human fetal atrial tissue, but not in adult hearts [158]. p21 was also found in developing rat ventricular myocytes [159]. In both studies, no comparison with old ages was made. Some p21-expressing cardiomyocytes were detected in E15.5 developing mouse embryos [160] and in trabecular myocardium at E18.5 [161]. The number was largely increased in Foxm1 knockout embryos as well as in Tbx20 overexpressing hearts at the early stages of development, which correlated with reduced proliferation and cardiac hypoplasia [160,162,163]. Fog-2 was described as a direct transcriptional repressor of p21 in cardiac development. Fog-2 mutant embryos showed multiple cardiac malformations, upregulation of p21, and thin-walled myocardium [164]. p21 expression had also been described in developing skeletal muscle, bones, lung, and spinal cord [165,166,167,168,169]. p21 has been also implicated in the mitotic arrest in male mouse germ cells during embryonic development [170]. An elegant study analyzing p21 and P57 double-mutant mice showed that both proteins redundantly control differentiation of skeletal muscle, bones, and alveoli in the lungs. Mice lacking both p21 and p57 failed to form myotubes, and displayed enhanced proliferation and apoptotic rates of myoblasts clearly indicating a role of p21 and P57 in normal muscle development [171]. Skeletal defects were more pronounced in embryos lacking p21 [171]. This report clearly shows the redundancy of the different proteins in cell cycle control and might explain the only few phenotypes observed in single knockout animals despite the importance of the cell cycle regulators for embryonic development.

**Figure 1:**
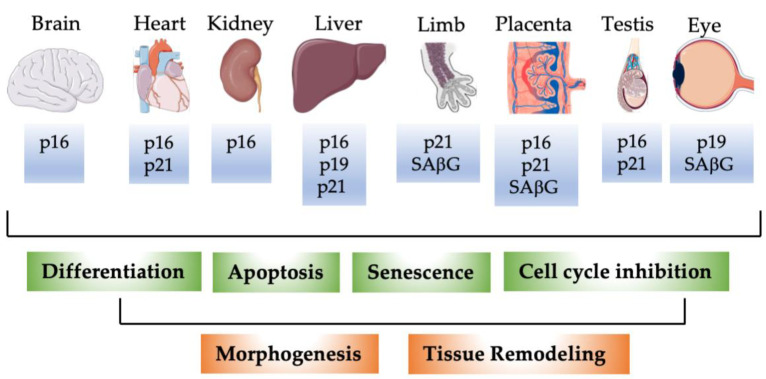
Schematic illustration of detection of p16INK4A, p14ARF/p19ARF, p21, and SAβG in selected murine organs during development. P16: p16INK4A; p19: p14ARF/p19ARF. During development, p16INK4A, p14ARF/p19ARF, p21, and SAβG not only mark senescent cells. p16INK4A, p14ARF/p19ARF, and p21 proteins are expressed in distinct cell types during different developmental stages. Expression of the individual proteins correlates with lower expression of proliferation markers in agreement with their classical function as cell cycle inhibitors, with apoptosis, and cellular differentiation. These fundamental processes contribute dynamically to tissue remodeling and morphogenesis during embryonic development.

Besides these studies implicating mostly p21 in embryonic development and differentiation, several reports also pointed to senescence as a potential mechanism for normal embryonic development. Munoz–Espin and colleagues performed whole-mount senescence-associated β-galactosidase SaβG) staining in mouse embryos. They detected SaβG activity in endolymphatic sacs of the developing ear, in the closing neural tube, the apical ectodermal ridge (AER) of the limbs, and later in regressing interdigital webs, around the vibrissae, and in the mesonephros of dissected gonad-mesonephros complexes [13]. However, in the dissected gonad-mesonephros picture of the manuscript, some SAβG staining also seems to be visible in the gonad and the Wolffian/Muellerian duct system. In further analyses, the authors focused on the endolymphatic sac and the mesonephros. SAβG activity in regressing mesonephros had been reported already earlier in chicken embryos [172]. SAβG activity was also detected in mesonephros and endolymphatic sacs of human embryos around 9 weeks of development [13]. As a marker of proliferation, they used Ki67 staining and found low proliferation in cells with SAβG activity. Nevertheless, during several developmental time points, some Ki67-positive cells were still detectable in SAbG-positive structures. As a major actor in developmental senescence, the authors suggested p21 based on immunostainings for several markers of senescence in endolymphatic sacs and mesonephros. Interestingly, the authors detected high p16INK4A expression in the gonad, which was not further commented upon. SAβG-positive cells were surrounded by macrophages and disappeared during ongoing development while the macrophage infiltration persisted longer. The attraction of macrophages was attributed to the SASP of SAβG-positive cells, which resulted in the now widely accepted concept that senescent cells secrete a cocktail of molecules, which beside other effects attract macrophages that finally clear the senescent cells [13,173,174,175,176]. A subset of p16INK4A expressing macrophages, which are SAβG-positive and might mediate this effect was identified recently in mouse tissues [177]. However, as Munz–Espin and colleagues immunostained the embryos also for p16INK4A, the macrophages in their model might represent a distinct population. Also, in tumor-bearing mice, doxorubicin induced senescence and a SASP in the skin, independent of macrophages and neutrophils [178], suggesting a certain variability in the events of senescent cell clearance. Finally, Munz–Espin investigated potential developmental defects in p21-deficient embryos. p21 knockout embryos had abnormal endolymphatic sacs with infoldings at late stages of development (E18.5), which disappeared after birth most likely due to macrophage clearance. Also in this case, the developmental program to remove the abnormal cells was independent of SAβG-positive cells or p21. In the uterus, which partially derives from the regressing Wolffian duct, the authors observed frequent septation and consequently a lower number of pups in p21 knockout mice [13], a phenotype, which had been missed in the first global analyses of these animals. Storer et al. used in parallel a similar approach and detected SAβG-positive cells in the AER, otic vesicle, the eye, branchial arches, gut endoderm, neural tube, tail, gall bladder, and interdigital tissue [179]. Similarly, in this report, p16INK4A and p14ARF/p19ARF seemed not to be involved in embryonic senescence, but p21 knockout embryos displayed less SAβG-positive cells. Instead of becoming senescent, cells underwent apoptotic cell death and were cleared by macrophages. Interestingly, the mesenchyme directly below the AER showed reduced proliferation indicating that developmental senescence is directly linked to cell proliferation and patterning of neighboring structures [179]. As additional sites of SAβG-staining, the developing bones, placental trophoblast cells [180], and the visceral endoderm [181] were identified. In the case of the visceral endoderm, SAβG-staining was not indicative of senescence [181]. Senescent cells have been described in a variety of developing organisms including birds, zebrafish, axolotl, naked mole rats, xenopus, mouse, and humans [13,172,179,182,183,184,185,186,187,188], mostly on the basis of SAbG-staining. More recently, the utility of SAβG-staining for the detection of developmental senescence has been questioned as also apoptotic cells, e.g., in the interdigital tissue and postmitotic neurons are stained independent of senescence [189,190,191]. Additionally, SAβG and p16INK4A expression have been shown to be induced in macrophages in response to physiological stimuli, without the cells being senescent [192]. Furthermore, we described recently p16INK4A expression at different developmental time points and several organs, which did not correspond to the known sites of SAβG expression. Only in old animals, but not during development, was a significant correlation between p16INK4A expression and SASP factors detectable. Of interest is also the detection of senescence cells and significant SASP activation in the development and response to stress in naked mole rats, which are considered a model of cancer-free longevity [186]. Reported sites of SAβG-staining, p16INK4A, p19p14ARF/p19ARF, and p21 expression during development are briefly summarized in Table 1 and illustrated in Figure 1.

## 4. p16INK4A, p14ARF/p19ARF, and p21 in Homeostasis

The implications of p16INK4A, p14ARF/p19ARF, and p21 in senescence and aging are extensively investigated and reviewed elsewhere [4,5,6,11,56,194,195,196,197,198,199]. Senescence has long been considered an important mechanism to prevent tumorigenesis, thus acting as a guardian of homeostasis, which agrees with p16INK4A, p14ARF/p19ARF, and p21 knockout mouse models. However, more recent data allow to draw a more differentiated picture of senescence and the SASP in tumor initiation and progression (reviewed in [200,201,202,203,204]). Organ and tissue homeostasis, however, do not only play a role in cancer prevention, but represent the central organizing principle of physiology and pathophysiology [205]. Major homeostatic and pathophysiological processes involving p16INK4A, p14ARF/p19ARF, and p21 are summarized in Table 2 and described below.

### 4.1. p16INK4A

Maintenance of cardiac function during aging and cardiac remodeling had to some extent been attributed to the expansion and differentiation of cardiac-resident stem cells (reviewed in [197]). To which extent cardiac stem and progenitor cells contribute to myocytes, endothelium, smooth muscle cells, etc., in cardiac repair is still a matter of debate [111,117,197,231,232,233,234]. In contrast to earlier publications, it is now widely accepted that cardiac, but not hematopoietic-derived progenitor cells are implicated in the cardiac repair [235]. With increasing age, the fraction of p16INK4A-expressing cardiac stem cells and expression of SASP factors increased in human biopsies [78]. A fraction of SAβG-negative cardiac stem cells improved cardiac function after experimental myocardial infarction in immunosuppressed mice while the fraction of SAβG-positive cells did not [78]. Notably, injection of the SAβG-positive cells did not worsen cardiac function after experimental myocardial infarction, which contrasts with the title of the manuscript [78]. The combination of the senolytic drugs dasatinib and quercetin as well as the elimination of p16INK4A-positive cells in the INK-ATTAC mouse model improved some cardiac parameters [78]. Unfortunately, neither the number of p16INK4A-positive cells nor cardiac function was determined in this set of experiments. As the values in INK-ATTAC mice and dasatinib and quercetin-treated animals differed for most parameters, it is possible that the cocktail of senolytic drugs has additional effects besides the elimination of p16INK4A-expressing cells. Of note, the original paper describing the generation and characterization of INK-ATTAC mice [72] reported a lack of INK-ATTAC induction in the heart, liver, and aorta, making it likely that the observed beneficial effects are due to secondary paracrine (SASP) induced events. In this original mouse model, time course studies showed that the elimination of p16INK4A expressing cells reflects the attenuated progression of age-related declines rather than a reversal of aging [72]. This seems to be somehow in contrast to the study mentioned before [78]. Most of the original investigations were done in the BubR1^H/H^ progeroid mouse genetic background, which might be slightly different from aged mice. In a following manuscript, the same group detected increasing p16INK4A expression in aged mice in all organs, but induction of the transgene with AP20187 did not affect the colon or liver expression of senescence markers [73]. However, heart and kidney morphological and expression parameters were to some extent normalized in aging INK-ATTAC mice treated with AP20187 and healthy lifespan extended. The shortest survival was measured in C57 wild-type mice treated with AP20187 [73]. In the heart, cardiomyocyte diameters were reduced in aging INK-ATTAC mice treated with AP20187, while the left ventricular wall thickness as an alternative measure of hypertrophy was unaffected suggesting that the clearance in INK-ATTAC mice is partial and tissue-selective [73]. This transgenic mouse model under the control of a 2.6 kB p16INK4A-promoter fragment might not completely reflect endogenous p16INK4A expression and regulation as we detected p16INK4A expression in the heart and liver [38,39] and elimination of p16INK4A expressing cells in the p16INK4ACre;DTA model caused cardiac and liver fibrosis and reduced health span [39], which is in agreement with the notion that senescent cells contribute to tissue repair and maintenance [211,221].

Elevated expression of endogenous p16INK4A has been recently demonstrated in a myocardial infarction (MI) model in mice. Forced overexpression of p16INK4A improved cardiac function while silencing of p16INK4A deteriorated cardiac function. As a possible underlying mechanism, reduced fibroblast proliferation, and collagen accumulation and less cardiac fibrosis was attributed to the classical cell-cycle inhibitory function of p16INK4A [213]. Increased cardiomyocyte proliferation and better functional recovery after MI has been reported in p16INK4A knockout mice [144]. This discrepancy remains currently unresolved.

Genome-wide association studies have implicated the human p16INK4AInk4a/Arf locus in the risk for cardiovascular and metabolic diseases and type 2 diabetes mellitus [236,237,238]. Deletion of a homologous region in mice caused reduced expression of p16INK4A and Cdkn2b, increased tumor incidence, and increased body weights and mortality in the animals [88]. Knockdown of *p16INK4A* enhanced adipogenesis in vitro, and adipose tissue formation especially in the pericardial fat was enhanced in *p16INK4A* knockout mice [208]. The role of p16INK4A in adipogenesis seems to be related via several molecular mechanisms to PPAR gamma (reviewed in [214]). Senolytic drug treatment or the use of INK-ATACC mice has been shown to alleviate metabolic and adipose tissue dysfunction, improve glucose tolerance, enhance insulin sensitivity, lower circulating inflammatory mediators, and promote adipogenesis in obese mice [239]. p16INK4A regulates adipogenesis and adipose tissue insulin sensitivity mainly via CDK4 [208,240,241]. Part of the action of p16INK4A in adipose tissue is related to obesity-induced inflammation and immune cell polarization [228,242]. Bone marrow-derived macrophages from *p16INK4A* knockout mice show polarization towards an anti-inflammatory M2 phenotype and silencing of p16INK4A in macrophages from obese patients equally shifts the phenotype towards M2 macrophages [227,228]. These effects seem to be independent of proliferation and senescence [214], although earlier data indicated a critical role of the p16INK4A locus in proliferation and programming of progenitor cell populations [243]. Besides the effects of p16INK4A on macrophage polarization in adipose tissue, also increased white-to-brown adipocyte conversion associated with enhanced energy expenditure and insulin sensitivity has been reported in *p16INK4A* knockout mice [215]. Whether this is due to enhanced direct conversion from white to brown adipocytes or it results from enhanced differentiation of progenitor cells remains an open question.

In contrast to the results described above for the INK-ATACC model, which eliminates p16INK4A expressing cells, a transgenic “Super-Ink4/Arf” mouse model with slightly increased p16INK4A RNA expression in the liver has been described [219]. Despite one extra copy of p16INK4A, the animals showed no significant increase in p16INK4A protein expression in the liver, heart, muscle, or pancreatic islets. Nevertheless, they did not develop glucose intolerance with age and showed a higher insulin sensitivity. The authors argued that the small increases in p16INK4A are causing this protective effect against the development of age-related diabetes mellitus [219]. Increasing p16INK4A expression with age in pancreatic islets has been described. Forced overexpression of p16INK4A reduced islet proliferation, while old mice lacking p16INK4A in pancreatic islets demonstrated enhanced islet proliferation and survival after beta-cell ablation, which agrees with the “classical” antiproliferative effect of p16INK4A [220]. Several additional publications implicated p16INK4A in insulin secretion and beta-cell proliferation [79,216,244,245]. In addition, p16INK4A deficiency enhances fasting-induced hepatic glucose production via activation of PKA-CREB-PGC1α signaling [246]. Accumulation of senescent cells during aging promotes hepatic fat accumulation and steatosis via reduced capabilities of mitochondria to metabolize fatty acids. Elimination of senescent cells in INK-ATTAC mice or by treatment with a combination of the senolytic drugs dasatinib and quercetin reduces hepatic steatosis [17], while specific elimination of p16INK4A-expressing liver sinusoidal endothelial cells induces hepatic fibrosis and premature death [39]. In humans with loss-of-function mutations in CDKN2A encoding p16INK4A and p14ARF, carriers showed increased insulin secretion, impaired insulin sensitivity, and reduced hepatic insulin clearance. There were no significant differences between patients with mutations affecting both p16INK4A and p14ARF and subjects with mutations affecting p16INK4A only suggesting that these effects are indeed due to the p16INK4A loss of function [217]. Taken together, the different reports from mice and humans suggest that p16INK4A acts at multiple levels of glucose homeostasis and metabolism especially in older individuals. Potential developments of therapeutic strategies for type 2 diabetes mellitus by modifying p16INK4A should be considered with care given the potential cancer risk.

Besides the described implications of p16INK4A in the cardiovascular system, adipose tissue, and metabolism, several publications also identified potential functions in the circadian clock [247], neurogenesis, neuronal trans-differentiation, and axon regeneration [222,248,249,250], most of them in agreement with cell cycle control by p16INK4A.

In an elegant study, Demaria and colleagues identified senescence as a potential adaptative mechanism for tissue repair. They generated a bacterial artificial chromosome (BAC)-transgenic mouse line containing 50 kb of the genomic region of the p16INK4A locus, a luciferase and red fluorescent protein (RFP) reporter, and a truncated herpes simplex virus 1 (HSV-1) thymidine kinase (HSV-TK) cassette allowing the elimination of cells with activated p16INK4A locus upon treatment with ganciclovir [41]. RFP-positive cells showed increased SAβG staining and increased levels of mRNAs encoding p16INK4A, p21, and the SASP factors IL-6, MMP-3, and VEGF, but not IL-5, suggesting that the RFP-marked cells are indeed senescent. The elimination of these cells caused delayed cutaneous wound healing. A similar phenomenon was also observed in p16INK4A/p21 double knockout mice, which do not show senescence [251] but not in single p16INK4A or p21 knockout animals, which are able to compensate the lack of one protein by the other in terms of senescence [41,251]. As major p16INK4A-positive cell types in the cutaneous injury model, endothelial cells and fibroblasts were identified [41], which agrees with our recent observations [38,39]. Senescent endothelial cells and fibroblasts appear early after injury and accelerate wound closure by inducing myofibroblast differentiation through the secretion of platelet-derived growth factor AA [41]. Using the same mouse model, several reports indicated that the removal of p16INK4A-expressing cells attenuated post-traumatic osteoarthritis [225], had no effect on age-related bone loss [206], prevented age-related intervertebral disc degeneration [23], improved irradiation-induced immune cell functional decline [229,230], protected cognitive function [223], and alleviated cisplatin-induced peripheral neuropathy in mice [224]. Senescent cells might also contribute to the release of hemostasis-related factors, which in excess might contribute to thromboembolic events in the old [252]. Most recently, the mouse model was used to study cellular senescence in cigarette smoke-induced lung injuries in adult and old mice [253]. Cigarette smoke induced senescence, p16INK4A, and p21 expression in adult animals, though surprisingly the opposite was observed in old animals [253].

In line with the role of p16INK4A in cardiovascular progenitor cells mentioned above, a potential function was postulated in skin stem and progenitor cells [254] and a higher colony-forming ability and replating efficiency measured in bone marrow-derived progenitor cells from p16INK4A knockout mice [255], which has been reviewed elsewhere [105,256,257]. In aged p16INK4A knockout mice, superior repopulating ability in bone marrow transplantation experiments compared with wild-type animals was noted, while only tiny differences were detectable under baseline conditions [258]. In mice with tetracycline-inducible overexpression of a human p16INK4A transgene, proliferation of intestinal stem cells was diminished, and animals showed signs of accelerated aging, which were mostly reversible upon withdrawal of tetracycline [207]. In this model, p16INK4A overexpression was not associated with senescence as evidenced by lack of SAβG staining. In contrast to these mouse models, to the best of our knowledge, neither major skin nor hematopoietic nor intestinal stem cell abnormalities were reported in patients with p16INK4A mutations.

### 4.2. p14ARF/p19ARF

Although p16INK4A and p14ARF/p19ARFArf are transcribed from the same locus, the proteins have some overlapping as well as distinct functions. Mice with an extra copy of Ink4/Arf or the downstream effector P53 showed resistance against cancer, which is in line with the general cell cycle and tumor suppressor function [259,260,261,262]. Intercrosses of both mouse lines showed additional resistance to cancer and extended longevity [209]. It is likely that the extended longevity in this model is at least in part due to the preservation of the stem cell pool in different organs [209,263,264,265,266]. Extra copies of Ink4/Arf in homozygous mice induced delayed aging, reduced the cancer incidence, improved longevity, diminished kidney lesions, and DNA damage, but also caused male infertility [210]. Different mouse models with activated P53 signaling also showed resistance to cancer development, but decreased the lifespan and premature onset of age-related diseases such as osteoporosis and tissue atrophy [267,268]. In line with this, these mouse models present reduced hematopoietic, mammary gland, neuronal, and pancreatic stem and progenitor cells with impaired hematopoiesis, mammary atrophy, decreased olfaction, and disturbed glucose homeostasis [269,270,271,272]. Whether the discrepancies in the longevity of the various mouse models are due to different levels of activation of the Arf-P53 pathway remains elusive. Taken together, the p14ARF/p19ARFArf-p53 pathway seems to be mostly responsible to maintain the stem cell pool and promote homeostasis, while data mostly from the transgenic p16INK4A-INK-ATTAC and p16INK4A-3MR [41,239] mouse models suggest that elimination of p16INK4A might be beneficial for homeostasis and healthy aging although this view was challenged recently [39].

### 4.3. p21

Recently, two mouse models were established to specifically address the role of p21 in senescence and tissue homeostasis. The first consists of an inducible p21-Cre model (CreERT2), which allows after crossing with different floxed mice monitoring or elimination of p21 expressing cells [212]. The second mouse strain is comparable to the p16INK4A-INK-ATTAC mouse model but uses a 3.2 kb p21 promoter fragment driving expression of the FKBP–Caspase-8 fusion suicide protein. The construct was inserted in the Rosa26 locus [226]. The p21-CreERT2 mice were crossed with a luciferase reporter, and luminescence was measured in vivo after doxorubicin treatment or a high-fat diet as known inducers of senescence. Next, p21-CreERT2 animals were crossed with floxed knock-in tdTomato mice confirming the expected increase in fluorescent cells in several organs in old mice. Finally, the p21-CreERT2 line was crossed with a DTA ablator line, and physical fitness was measured in old mice treated with Tamoxifen and controls. The elimination of p21-positive senescent cells increased walking speed, grip strength, hanging endurance, daily food intake, and daily activity indicating a rejuvenation phenotype in response to the elimination of p21-expressing cells [212]. Surprisingly, p16INK4A- and p21-expressing cell populations seem to be different [212], which is contrasting with the lack of senescence in p16INK4A/p21 double knockout animals [251]. Also in the p21–ATTAC model, the clearance of p21- but not p16INK4A-positive senescent cells prevented radiation-induced osteoporosis and bone marrow adiposity [226], supporting the view that p16INK4A- and p21-dependent senescence comprise different and independent pathways [3,5,22,273]. A high number of p21- but not p16INK4A-expressing cells was detected in visceral adipose tissue of obese mice, mostly preadipocytes, endothelial cells, and macrophages [218]. In contrast to visceral adipose tissue, the heart, kidney, liver, and brain of old mice express high levels of p16INK4A in endothelial cells [38,39]. Elimination of p21-expressing cells using the p21-CreERT2 line crossed with the DTA ablator line worked in preadipocytes, macrophages, and leukocytes, but not in the endothelial compartment. Functionally, it improved glucose homeostasis and insulin sensitivity in obese mice. Interestingly, the removal of p21-positive cells had less metabolic benefits in female than male mice [218] adding one more layer of complexity to potential translational approaches. Of note, the senolytic cocktail of dasatinib plus quercetin was able to remove p21-positive senescent adipocytes but not endothelial cells and macrophages [218]. Nevertheless, it improved glucose homeostasis and insulin sensitivity and reduced pro-inflammatory SASP secretion [218]. Although this elegant study clearly supports the idea of senolytic drugs as a therapeutic strategy for obesity-induced metabolic dysfunction, it also raises new questions about the mode of action of the senolytic drug cocktail, which seems to target one specific senescent cell type.

A recent elegant study showed that in response to cellular stress, p21 and p16INK4A are upregulated. Both induce cell cycle arrest and SASP expression, but the SASPs are different [274]. The p21-induced secretome is characterized by the release of additional immunosurveillance factors, in particular Cxcl14, which are lacking in the p16INK4A-induced SASP. Consequently, the p21-induced secretome attracts macrophages. At later stages, the macrophages polarize into a M1 phenotype, and the p21-exressing cells are cleared via T cells. Most importantly, the authors showed that the p21-induced SASP places the cells under immunosurveillance and establishes a timer mechanism for the cell fate. In the case of p21, the expression normalizes within 4 days in mice, macrophages withdraw, and the cells are not cleared. Thus, the specific p21-induced SASP sets the time frame for the switch between surveillance and cell clearance mode of the immune system [274]. This mechanism might contribute to the developmental decisions described above, where individual cells are mostly characterized by transient expression of p21.

## 5. Open Questions and Perspectives

The establishment of several p16INK4A- and p21-deleter mouse lines mentioned above contributes largely to our understanding of senescence and aging phenotypes. As both proteins are expressed in different cell types and ablation has diverse effects, senescence is not one biological entity, but comprises different cellular events and divergent SASPs. The picture might be even more complex considering that in a given cell type aging is heterogenous [275] and tissues are in different stages of senescence [276,277]. The observation of beneficial effects in organs where the transgene is not expressed in p16INK4A-INK-ATTAC mice suggests a major role of SASP normalization instead of direct elimination of senescent cells. This is further supported by the recent p21-Cre line data [218] and the fact that the SASP from a small number of cells is sufficient to induce senescence in young mice and senolytic drugs induced a rejuvenation phenotype [278]. The next complicating issue is that the SASP is also not a homogenous cocktail of released factors but might highly differ in the composition of immunomodulatory factors and thus determine more physiological aging versus pro-inflammatory deteriorating phenotype (reviewed in [3,279]). Interestingly, different p16INK4A-positive cell elimination mouse models showed diverse phenotypes with the p16INK4A-INK-ATTAC model delaying aging phenotypes and increasing lifespan [72], while in the p16INK4A-3MR model wound healing was disturbed [41], and in p16INK4ACre;DTA mice liver fibrosis and reduced health-span were observed [39]. Thus, it would be important to determine whether p16INK4A-expressing cells are the same in the three models under baseline conditions. For this purpose, our recently established and knockout-validated immunohistochemistry protocol could be a useful tool [38]. As p16INK4A expression is not an off–on phenomenon, but increases from embryonic stages until old age [38], in the next step it would be interesting to determine whether p16INK4A-expressing cells in the mouse models are eliminated at different levels of p16INK4A expression. If this is the case, sorting of the cells and secretome analysis could define the secretory phenotype of protective versus detrimental p16INK4A expressing cells which finally may serve as a rejuvenation approach in aged patients without the need and limitations of overexpression of reprogramming factors [279].

## Figures and Tables

**Table 1:** Detection of senescence markers during development.

Localization	Detected Signal	Species	Ref.
Heart, kidney, brain, liver	p16INK4A, p14ARF/p19ARF, p21 mRNA, p16INK4A protein	mouse	[38]
Brain	p16INK4A mRNA	rat	[125]
Olfactory epithelium	p16INK4A, p14ARF/p19ARF, p21 protein	mouse	[126]
Hematopoietic stem cells	p16INK4A, p14ARF/p19ARF mRNA	mouse	[127]
Chondrocytes, osteoblasts	p16INK4A, p21 protein	mouse	[128]
Male germ cells	p16INK4A, p21 mRNA	mouse	[129,170]
Endometrium in early pregnancy	p16INK4A mRNA, p16INK4A protein	mouse	[131]
Endometrium in pregnancy	p16INK4A protein	human	[133]
Syncytiotrophoblast	p16INK4A, p21 protein	human	[182]
Intestinal stem cells	p16INK4A protein	mouse	[134]
Perivascular ocular cells	p14ARF/p19ARF protein	mouse	[147,149,150,151,152]
Hepatoblasts	p14ARF/p19ARF, p21 protein	mouse	[156]
Heart	p21 protein	human, rat, mouse	[158,159,160,161]
Muscle, cartilage, skin, nasal epithelium, hair follicles	p21 mRNA, p21 protein	mouse	[165,166,167,171]
Mesonephros	SAβG	bird	[172]
Endolymphatic sacs, mesonephros	SAβG	mouse, human	[13]
Neural tube, AER, vibrissae	SAβG	mouse	[193]
AER, otic vesicle, eye, branchial arches, gut endoderm, neural tube, tail, gall bladder, and interdigital tissue	SAβG	mouse	[179]
Bones, placenta trophoblast cells	SAβG	mouse	[180]
Visceral endoderm	SAβG	mouse	[181]
Inner ear	SAβG	birds	[183]
Pronephros, cement gland, oral cavity, olfactory epithelium, lateral organs, gums	SAβG	axolotl	[184,185]
Yolk sac epithelium, lower part of the gut	SAβG	zebrafish	[185]
Nail bed, dermis, hair follicle, nasopharyngeal cavity	SAβG	Naked mole rat	[186]

Abbreviations: Ref.—Reference, AER—apical ectodermal ridge, SaβG—senescence-associated beta galactosidase

**Table 2:** Major phenotypes associated with p16INK4A, p14ARF/p19ARF, or p21 modifications in homeostasis and pathophysiology.

Pathophysiology/Homeostatic Mechanism	Intervention/Model	Outcome	Ref.
Physiology			
Age-related cardiomyocyte hypertrophy	INK-ATTAC mouse	Cardiac cell size↓	[73]
Age-related lipodystrophy	INK-ATTAC mouse	Adipose tissue mass ↑	[73]
Health-span	INK-ATTAC mouse	Survival ↑	[73]
Health-span	p16INK4ACre; DTA	Survival ↓	[39]
Age-related bone loss	p16INK4A-3MR mouse	=	[206]
Aging-related intervertebral disc degeneration	p16INK4A-3MR mouse	Histological disc morphology improved	[23]
Aging features	p16INK4A overexpression	Accelerated	[207]
Adipocyte formation	p16INK4A−/−	Adipogenesis ↑	[208]
Longevity	p16INK4A−/−, p14ARF/p19ARF−/−, P53−/−	Lifespan ↑	[209]
Longevity Male fertility	p16INK4A/p14ARF/p19ARF overexpression	Lifespan ↑ Absence of sperm	[210]
			
Lifespan	INK-ATTAC mouse, BubR1^H/H^ background	=	[72]
Physical fitness	INK-ATTAC mouse BubR1^H/H^ background	Endurance ↑	[72]
Aging-associated liver fibrosis	p16INK4ACre; DTA	Fibrosis ↑	[39]
			
Aging-associated hepatic steatosis	INK-ATACC mouse	Fat accumulation ↓	[17]
			
Wound healing	p16INK4A-3MR mouse	Wound closure ↓	[41]
			
Wound healing	CCN1-dependent p16INK4A induction	Fibrosis ↓	[211]
			
Aging-associated glomerulosclerosis	INK-ATTAC mouse	Glomerulosclerosis ↓	[73]
			
Aging-related physical activity loss	p21Cre;DTA	Physical fitness ↑	[212]
			
Sarcopenia	INK-ATTAC mouse, BubR1^H/H^ background	Sarcopenia delayed	[72]
			
Glaucoma	INK-ATTAC mouse, BubR1^H/H^ background	Glaucoma onset delayed	[72]
**Pathophysiology**			
Myocardial infarction	INK-ATTAC mouse, senolytics	Cardiomyocyte proliferation ↑	[78]
			
Myocardial infarction	p16INK4A overexpression	Cardiac function ↑ Fibrosis ↓	[213]
			
Myocardial infarction	p16INK4A−/−, p14ARF/p19ARF−/−	Cardiomyocyte proliferation ↑ Cardiac function ↑	[144]
			
Obesity	INK-ATACC mouse	Insulin sensitivity ↑ Metabolic dysfunction ↓	[214]
			
Adipocyte conversion	p16INK4A−/−	White to brown ↑	[215]
			
Diabetes	p16INK4A overexpression	Insulin secretion ↑	[216]
			
Glucose homeostasis	Human p16INK4A inactivating mutations	Insulin secretion ↑ Insulin sensitivity ↓	[217]
			
Glucose homeostasis Insulin sensitivity in obese mice	p21Cre;DTA	GTT ↑ ITT ↑	[218]
			
Diabetes	p16INK4A overexpression	Insulin sensitivity ↑ Metabolic dysfunction ↓	[219]
			
Pancreatic beta cell regeneration	p16INK4A overexpression	Islet proliferation ↓	[220]
			
Pancreatic beta cell regeneration	p16INK4A−/−	Islet proliferation ↑	[220]
			
Liver fibrosis	p53−/−; p16INK4A/p14ARF/p19ARF−/−	Fibrosis ↑	[221]
			
Ionizing radiation-induced reduction of neurogenesis	p16INK4A−/−	partial restoration	[222]
Radiation-induced impairment of cognitive function	p16INK4A-3MR mouse	Cognitive function ↑	[223]
Cisplatin-induced peripheral neuropathy	p16INK4A-3MR mouse, senolytics	Neuropathy ↓	[224]
Post-traumatic osteoarthritis	p16INK4A-3MR mouse	Osteoarthritis ↓	[225]
Radiation-induced osteoporosis	p21INK-ATTAC mouse	Osteoporosis ↓	[226]
			
Macrophage polarization	p16INK4A−/−	Anti-inflammatory phenotype ↑	[227]
Macrophage polarization	Human p16INK4A silencing	Anti-inflammatory phenotype ↑	[228]
			
Irradiation-induced immune dysfunction	p16INK4A-3MR mouse	T-cell proliferation ↑ Macrophage phagocytosis ↑	[229,230]

↑: Higher, ↓: Lower, =: not significantly different, −/−: knockout mouse model, BubR1^H/H^: mouse model of accelerated aging with multiple age-related pathologies, INK-ATTAC mouse: allows deletion of p16INK4A expressing cells, p16INK4ACre;DTA: mouse model allows deletion of p16INK4A expressing cells, p16INK4A-3MR mouse: allows deletion of p16INK4A expressing cells, GTT: glucose tolerance test, ITT: insulin tolerance test
